# A GAN-CNN Fusion Framework for Deep Learning-Based DOA Estimation in Low-SNR Environments

**DOI:** 10.3390/s26051676

**Published:** 2026-03-06

**Authors:** Zhenshan Zhang, Wenjie Xu, Haitao Zou, Shichao Yi

**Affiliations:** 1 School of Computer Science and Engineering, Jiangsu University of Science and Technology, Zhenjiang 212003, China; 231210702119@stu.just.edu.cn (Z.Z.); 231210701126@stu.just.edu.cn (W.X.); htzou@just.edu.cn (H.Z.); 2 School of Science, Jiangsu University of Science and Technology, Zhenjiang 212003, China; 3 Zhenjiang Jizhi Ship Technology Co., Ltd., Zhenjiang 212003, China

**Keywords:** DOA estimation, deep learning, generative adversarial network, array signal processing, data augmentation

## Abstract

Direction of Arrival (DOA) estimation faces significant performance degradation under low Signal-to-Noise Ratio (SNR) conditions, where traditional algorithms and deep learning models struggle due to corrupted spatial information and limited training data. To address these challenges, this paper introduces a novel two-stage framework that integrates a Generative Adversarial Network (GAN) for signal enhancement with a complex-valued Convolutional Neural Network (CNN) for DOA estimation. The proposed GAN incorporates an attention mechanism and a dedicated phase-consistent loss function to suppress noise while preserving spatial phase information critical for accurate direction finding. Enhanced signals are transformed into covariance matrices and processed by a complex-valued CNN designed to extract robust spatial features. Extensive experiments demonstrate that the proposed method achieves a DOA accuracy of 72.2% and a Root Mean Square Error (RMSE) of 3.9° at —10 dB SNR with 500 snapshots, substantially outperforming conventional and deep learning baselines. The framework also shows strong robustness to limited data, maintaining 93.8% accuracy with only 50 snapshots. The framework offers a practical solution for reliable DOA estimation in low-SNR and data-scarce environments.

## 1. Introduction

DOA estimation represents a fundamental problem in array signal processing with widespread applications in radar systems, wireless communications, sonar, and acoustic signal processing. Accurate DOA estimation is crucial for spatial filtering, beamforming, and source localization in various civilian and military systems [1,2,3].

Traditional DOA estimation algorithms can be broadly categorized into phase-comparison techniques, subspace-based methods, and maximum likelihood approaches. Phase-comparison methods estimate the incident angle by measuring inter-sensor phase differences with relatively low computational cost, which makes them attractive for real-time and hardware-efficient implementations; however, they typically require careful calibration and ambiguity resolution when long baselines are used [4,5,6,7,8,9,10].

Classical subspace-based techniques such as Multiple Signal Classification (MUSIC) and Estimation of Signal Parameters via Rotational Invariance Techniques (ESPRIT) have demonstrated remarkable performance under ideal conditions. These methods exploit the eigenstructure of the covariance matrix to separate signal and noise subspaces. Nevertheless, their performance can become more sensitive to noise and sample limitations in low-SNR regimes or with a small number of snapshots, which may lead to reduced robustness in challenging environments [11,12,13]. Maximum likelihood methods, while statistically efficient, suffer from computational complexity that limits their practical implementation.

Recent advances in deep learning have inspired novel approaches to DOA estimation that circumvent limitations of traditional methods. CNNs have been successfully applied to learn spatial features directly from array data or covariance matrices, while Recurrent Neural Networks (RNNs) and their variants have been employed to capture temporal dependencies in signal sequences [14,15].

From a computational perspective, classical subspace-based methods typically involve covariance estimation and subspace separation, where eigendecomposition introduces non-negligible complexity, and MUSIC further requires a grid search over candidate angles, which increases runtime proportionally to the angular resolution. As a result, their time to first estimation (TTFE) is largely determined by the snapshot accumulation needed for a reliable covariance estimate as well as the subsequent subspace separation and spectral search.

In contrast, DNN-based techniques usually shift the main computational burden to the offline training stage. Once trained, inference can be executed via a single forward pass with fixed latency, making them attractive for real-time deployment, especially on GPUs. However, their TTFE still depends on the input representation: models that rely on covariance matrices require collecting sufficient snapshots before forming R, whereas end-to-end networks operating on raw snapshots can potentially reduce TTFE by producing estimates with fewer samples or in a streaming manner. Despite these advantages, most deep learning-based DOA estimators remain vulnerable to performance degradation in noisy environments, as they primarily learn mapping functions without explicit denoising mechanisms. Moreover, existing CNN/RNN-based models often focus on feature extraction from raw or preprocessed data while being sensitive to noise-induced phase distortion, which is indispensable for spatial parameter estimation [16,17].

In this work, the term real time refers to online operation with bounded and predictable latency, rather than implying a universal microsecond-level end-to-end response across all platforms. We distinguish (i) the algorithmic latency after the input representation is available and (ii) the TTFE, which additionally includes the data acquisition window. For covariance-based pipelines, TTFE is dominated by the snapshot accumulation required to form the sample covariance matrix. Let *K* be the snapshot number and $f_{s}$ be the effective acquisition rate; the observation window is

(1) $$T_{\mathrm{win}}=\frac{K}{f_{s}},$$

and the TTFE can be approximated as

(2) $$T_{\mathrm{TTFE}}\approx T_{\mathrm{win}}+T_{\mathrm{alg}},$$

where $T_{\mathrm{alg}}$ includes covariance construction and the subsequent inference/processing. Therefore, any absolute time value is scenario-dependent and should not be interpreted as a general definition of real-time DOA estimation. Notably, microsecond-level update rates reported by phase-interferometry/naive techniques are typically achieved via highly optimized hardware pipelines and per-snapshot processing, whereas covariance-based estimators trade longer observation windows for improved robustness under low SNR and limited data.

The integration of signal enhancement techniques with DOA estimation presents a promising direction to address noise robustness. Generative Adversarial Networks (GANs) have emerged as powerful tools for signal enhancement and denoising applications. In audio and speech processing, GAN-based enhancement has demonstrated remarkable capability in preserving signal integrity while suppressing noise. However, the application of GANs to array signal enhancement for DOA estimation has not been fully exploited, particularly concerning the preservation of spatial phase information critical for accurate direction finding [18].

The development of integrated enhancement-estimation frameworks presents several fundamental challenges that require careful consideration [19]. The primary challenge involves maintaining phase coherence during the denoising process to preserve spatial information, which is particularly crucial for DOA estimation as it fundamentally relies on precise phase relationships across array elements. Any phase distortion introduced during signal enhancement can severely degrade angle estimation accuracy and compromise the overall system performance. Another significant challenge lies in ensuring robust generalization capabilities across diverse noise types and varying SNR conditions, as practical operational environments often exhibit characteristics that may differ substantially from the training data distribution. This necessitates the development of algorithms that can adapt to unseen noise patterns and maintain consistent performance across a wide spectrum of signal quality conditions [20,21,22].

Furthermore, achieving an optimal balance between enhancement quality and computational efficiency represents a critical challenge, especially for real-time applications where processing latency and resource constraints impose practical limitations [23]. The enhancement process must deliver substantial noise suppression while maintaining computational tractability to enable deployment in resource-constrained environments. Additionally, the effective integration of enhancement and estimation modules within a unified framework poses substantial design challenges, requiring careful coordination between the two stages to ensure they work synergistically rather than as independent components. This integration must facilitate seamless information flow between modules while maintaining overall architectural coherence and optimization compatibility. These interconnected challenges collectively define the core technical obstacles that must be systematically addressed to realize effective integrated frameworks for DOA estimation in practical scenarios [24,25].

To address these challenges, this paper makes several key contributions that advance the state of the art in DOA estimation for low-SNR environments. We propose a novel two-stage framework that systematically combines GAN-based signal enhancement with CNN-based DOA estimation, creating an integrated architecture specifically engineered to operate effectively in challenging signal-to-noise ratio conditions. This comprehensive approach addresses the fundamental limitation of existing methods that struggle with performance degradation in noisy environments by incorporating a dedicated signal enhancement stage prior to the estimation process [26,27,28,29].

We develop an enhanced GAN architecture that incorporates sophisticated attention mechanisms and phase-consistent loss functions to preserve crucial spatial characteristics during the denoising process. The attention mechanism enables selective focus on temporally significant signal components, while the phase-consistent loss function ensures the preservation of phase information that is critical for accurate spatial processing and DOA estimation. This represents a significant improvement over conventional denoising approaches that often compromise phase integrity in pursuit of noise reduction.

Furthermore, we design a specialized complex-valued CNN architecture capable of effectively processing enhanced covariance matrices for accurate DOA classification. This network leverages the complex nature of array signals through dedicated processing pathways that maintain the rich information content in both real and imaginary components, enabling more effective feature extraction from the spatial covariance matrices that form the foundation of DOA estimation.

The remainder of this paper is structured as follows. Section 2 provides a comprehensive review of traditional DOA estimation methods and recent advances in deep learning-based approaches, along with an introduction to Generative Adversarial Networks. Section 3 details the proposed two-stage framework, including the design of the GAN-based signal enhancement module and the complex-valued CNN architecture for DOA estimation. Section 4 presents extensive experimental results and in-depth performance analysis under various conditions. Finally, Section 5 concludes the paper and suggests directions for future research.

## 2. Related Work

### 2.1. Traditional DOA Estimation Methods

Traditional DOA estimation methods can be broadly grouped into beamforming-based techniques, subspace-based methods, and sparse representation approaches [30,31]. Beamforming methods estimate DOA by scanning candidate angles and evaluating an output power criterion, offering relatively low implementation complexity but limited resolution under closely spaced sources or strong interference [32]. Subspace-based algorithms such as MUSIC and ESPRIT achieve super-resolution by exploiting the eigen-structure of the spatial covariance matrix; however, they are known to be sensitive to practical factors such as low SNR, limited snapshots, model mismatch, and source coherence, which can reduce robustness in challenging environments [33,34]. Sparse methods cast DOA estimation as a sparse recovery problem and can be effective with limited snapshots, but their performance depends on dictionary resolution and regularization choices [35].

Since the above principles are well established, we briefly emphasize a recent trend that is highly relevant to practical deployment: efficient real-time and hardware-oriented implementations of classical DOA/beamforming techniques. Recent works report FPGA/parallel architectures for MUSIC-like processing to reduce latency and resource usage, including dedicated acceleration of key blocks and high-level-synthesis (HLS) implementations. Processor-oriented ESPRIT implementations have also been proposed to enable scalable and efficient DOA estimation on digital hardware [36]. For beamforming, practical system-level implementations of adaptive methods on FPGA-based digital beamforming receivers have been investigated to meet real-time constraints [37]. In addition, phase-comparison AoA estimation has attracted renewed interest due to its hardware friendliness and reconfigurable full-digital architectures [38].

Despite these advances in efficient implementations, traditional pipelines remain fundamentally limited by their sensitivity in low-SNR and data-scarce regimes, motivating hybrid data-driven frameworks that explicitly enhance the received signals prior to DOA estimation. This motivates the enhancement–estimation paradigm adopted in this work.

### 2.2. Application of Deep Learning in DOA Estimation

The application of deep learning to DOA estimation represents a paradigm shift, leveraging neural networks to learn direct mappings from array data to source directions and thereby circumventing the stringent statistical assumptions of classical methods. Deep learning-based DOA estimators can be categorized by network architecture, input representation, and learning strategy, each suited to specific operational scenarios [39,40,41]. Among these, CNNs have been widely adopted due to their ability to extract spatial features from structured representations of array data. A common approach treats the spatial covariance matrix as a two-dimensional image, where the fundamental mapping learned by the network can be expressed as

(3) $$\hat{\theta }=f_{\mathrm{CNN}}\left(R\right),\;R=\frac{1}{K}\sum_{k=1}^{K}x\left(k\right)x^{H}\left(k\right)\in C^{N\times N}$$

with x(k) being the array snapshot at time *k* and *N* the number of sensors. To preserve the phase information critical for DOA, complex-valued CNNs have been developed that employ complex convolution operations. The output feature map can be written as

(4) $$Y=\sigma \;\left(W*X+b\right)$$

where ∗ denotes complex convolution, and W and b are complex-valued convolution kernels and bias terms, respectively. Here, σ(·) denotes a nonlinear activation function. In this work, we adopt a split activation applied independently to the real and imaginary parts, i.e.,

(5) $$\sigma \left(Z\right)=\phi \;\left(ℜ\{Z\}\right)+j\;\phi \;\left(ℑ\{Z\}\right)$$

where ϕ(·) is a standard real-valued nonlinearity (e.g., ReLU), ℜ{·} and ℑ{·} denote the real and imaginary components, and $j=\sqrt{-1}$.

For scenarios involving moving sources or sequential snapshots, recurrent architectures such as recurrent neural networks (RNNs) and Long Short-Term Memory (LSTM) networks are employed to capture temporal dependencies [42]. The state update and DOA prediction at time *t* are formulated as

(6) $$h_{t}=\mathrm{LSTM}\left(x_{t},h_{t-1}\right),\;{\hat{\theta }}_{t}=W_{o}h_{t}+b_{o}$$

where $h_{t}$ is the hidden state. Bidirectional RNNs further incorporate future context to improve accuracy, especially in offline batch processing [43]. A more recent trend emphasizes end-to-end learning frameworks that bypass explicit covariance computation altogether, mapping raw array data directly to DOA estimates

(7) $$\hat{\theta }=f_{\mathrm{CNN}/\mathrm{RNN}}\left(X_{\mathrm{raw}}\right),\;X_{\mathrm{raw}}\in C^{N\times T}$$

While eliminating the need for statistical preprocessing, such models demand large datasets and careful architectural design to implicitly learn spatial correlations.

To address the pervasive challenge of data scarcity, transfer learning techniques have been explored to adapt models trained on simulated data to real-world conditions [44]. This is typically achieved through composite loss functions that incorporate domain adaptation

(8) $$L_{\mathrm{total}}=L_{\mathrm{DOA}}+\lambda \cdot L_{\mathrm{domain}}\left(D_{\mathrm{sim}}D_{\mathrm{real}}\right)$$

Despite mitigating the simulation-to-reality gap, these approaches still struggle under low-SNR conditions or when confronted with unseen array geometries.

Notwithstanding significant progress, deep learning-based DOA methods continue to face several unresolved challenges, including pronounced sensitivity to noise, limited generalization across different array configurations, high computational costs for training complex networks, and a general lack of interpretability inherent to black-box models [45,46]. These limitations underscore the necessity for hybrid approaches that synergistically integrate established signal processing principles with the representational power of deep learning. The proposed GAN-CNN framework directly responds to this need by introducing a dedicated signal enhancement stage prior to estimation, thereby addressing the critical issue of noise robustness and paving the way for more reliable DOA systems in practical low-SNR environments.

### 2.3. GAN Model

Generative Adversarial Networks have been widely adopted in signal processing due to their adversarial learning mechanism [47,48,49]. A GAN consists of a generator (*G*) and a discriminator (*D*) trained in a minimax game:

(9) $$\min_{G}\max_{D}V\left(D,G\right)=E_{x∼p_{\mathrm{data}}}\left[\log D\left(x\right)\right]+E_{z∼p_{z}}\left[\log\left(1-D\left(G\left(z\right)\right)\right)\right]$$

where x denotes real/true samples drawn from the data distribution, and z is the latent input. In this work, each sample corresponds to complex-valued baseband array snapshots. To enable standard deep-learning operations, we represent complex snapshots by two channels, i.e., $x\in R^{2\times L}$, where *L* is the temporal length.

In signal enhancement settings, conditional GANs are particularly suitable because the generator learns a supervised mapping from noisy observations to clean targets:

(10) $$G:X_{\mathrm{noisy}}\to X_{\mathrm{clean}}$$

Here, $X_{\mathrm{noisy}}$ and $X_{\mathrm{clean}}$ are complex array-snapshot sequences represented by their real/imaginary channels. From a physical perspective, G(·) aims to suppress additive noise while preserving inter-sensor phase relations that encode the DOA information. In our implementation, 1D convolutions are applied along the temporal dimension to capture local time structures in the received signals, while the discriminator employs adaptive pooling to aggregate temporal evidence into a compact decision statistic for distinguishing enhanced signals from clean references [50,51,52,53].

Regarding array calibration, the training data are assumed to be obtained from a calibrated array model. To mitigate potential residual phase offsets after enhancement, a phase alignment step is applied before covariance construction in the preprocessing pipeline. Incorporating explicit sensor gain mismatch modeling via data augmentation or calibration-aware regularization is an important extension and will be considered in future work.

Our implementation builds upon the conditional GAN framework with key enhancements specifically designed for DOA signal processing requirements. The architecture incorporates multi-scale feature extraction through hierarchical downsampling blocks to capture both local and global signal characteristics. An integrated attention mechanism selectively emphasizes temporally significant components, while a symmetric decoder with skip connections ensures the preservation of critical phase information and temporal resolution.

The discriminator employs a computationally efficient design utilizing strided convolutions and adaptive pooling, maintaining strong discriminative capability while minimizing computational overhead. The training process incorporates a composite loss function $L=L_{\mathrm{adv}}+\lambda_{1}L_{L1}+\lambda_{2}L_{\mathrm{phase}}$ that combines adversarial training with magnitude reconstruction and phase consistency constraints. Notably, the phase consistency loss $L_{\mathrm{phase}}=E[|∠G\left(x_{\mathrm{noisy}}\right)-∠x_{\mathrm{clean}}{|}_{1}]$ explicitly addresses the preservation of spatial phase relationships essential for accurate DOA estimation.

Our implementation employs a two-phase training approach. First, generator pre-training uses only reconstruction losses ($L_{L1}+L\mathrm{phase}$) to provide stable initialization. Second, adversarial fine-tuning involves joint optimization of generator and discriminator with the complete loss function. This approach, combined with dynamic weight adjustment and gradient clipping, ensures stable training convergence and prevents mode collapse issues common in GAN training.

The proposed GAN framework demonstrates significant advantages for DOA signal enhancement by simultaneously addressing magnitude reconstruction and phase preservation, while maintaining computational efficiency suitable for real-time applications.

## 3. The Proposed Method

In this section, we present our proposed approach for DOA estimation in low-SNR environments using a novel two-stage deep learning framework. Traditional DOA estimation methods typically suffer from significant performance degradation under low-SNR conditions due to the corruption of signal subspace information in covariance matrices. This limitation affects both classical subspace-based algorithms and modern learning-based approaches, as noise contamination distorts the essential spatial features required for accurate direction finding. To address this fundamental challenge, we introduce an integrated framework that combines signal enhancement with deep learning-based DOA estimation. Our methodology employs an enhanced GAN architecture specifically designed for array signal denoising in the first stage, followed by a complex-valued CNN for robust DOA estimation in the second stage. The GAN-based enhancement module learns to recover clean signal characteristics from noisy inputs while preserving crucial phase information essential for spatial processing. The enhanced signals are then processed through a specialized CNN architecture that extracts discriminative features from covariance matrices for accurate angle estimation. By leveraging this sequential enhancement-estimation paradigm, our framework demonstrates superior robustness in challenging low-SNR scenarios while maintaining the computational efficiency required for practical implementations.

### 3.1. GAN Design for Signal Enhancement

#### 3.1.1. Network Architecture

The generator *G* employs an encoder–decoder architecture with attention mechanisms for processing complex-valued array signals. The input consists of noisy signals $X_{\mathrm{noisy}}\in R^{2\times L}$, where two channels represent real and imaginary components, and *L* is the sequence length. This representation preserves complex signal information while maintaining compatibility with standard deep learning frameworks.

**Figure 1:**
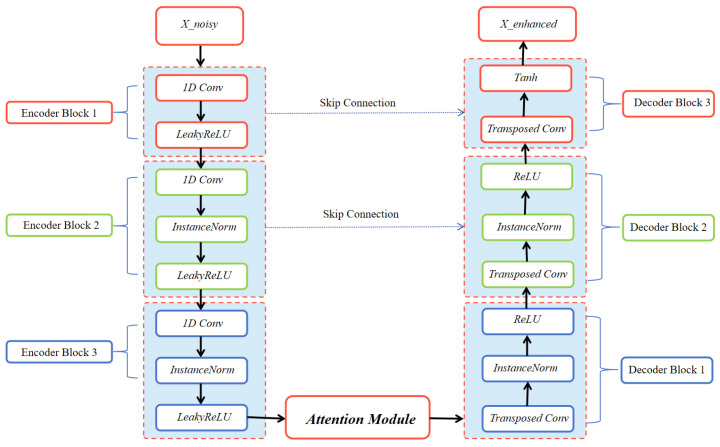
Detailed architecture of the generator network, illustrating the encoder–decoder structure with attention mechanism and skip connections for complex-valued signal processing.

The encoder pathway, shown in Figure 1, extracts multi-scale features through three downsampling blocks. The transformation can be expressed as:

(11) $$F_{1}=\mathrm{LeakyReLU}({\mathrm{Conv}1D}_{2\to 64}\left(X_{\mathrm{noisy}}\right))$$

(12) $$F_{2}=\mathrm{IN}(\mathrm{LeakyReLU}\left({\mathrm{Conv}1D}_{64\to 128}\left(F_{1}\right)\right))$$

(13) $$F_{3}=\mathrm{IN}(\mathrm{LeakyReLU}\left({\mathrm{Conv}1D}_{128\to 256}\left(F_{2}\right)\right))$$

where IN denotes instance normalization and strided convolutions reduce temporal resolution by half at each stage. An attention mechanism selectively emphasizes temporally significant components:

(14) $$F_{\mathrm{att}}=F_{3}⊗\sigma \left({\mathrm{Conv}1D}_{256\to 1}\left(F_{3}\right)\right)$$

where σ is the sigmoid function and ⊗ denotes element-wise multiplication. This enables focus on DOA-critical signal segments while suppressing noisy regions. The decoder performs symmetric upsampling with skip connections:

(15) $$D_{1}=\mathrm{IN}(\mathrm{ReLU}\left({\mathrm{ConvTranspose}1D}_{256\to 128}\left(F_{\mathrm{att}}\right)\right))$$

(16) $$D_{2}=\mathrm{IN}(\mathrm{ReLU}\left({\mathrm{ConvTranspose}1D}_{128\to 64}\left(D_{1}+F_{2}\right)\right))$$

(17) $$X_{\mathrm{enhanced}}=\tanh({\mathrm{Conv}1D}_{64\to 2}\left(D_{2}+F_{1}\right))$$

To make the mathematical description directly traceable to the generator blocks in Figure 1, we map each equation to its corresponding module: Equations (11)–(13) implement the three encoder/downsampling blocks (Enc-1/Enc-2/Enc-3) producing $F_{1}$, $F_{2}$, and $F_{3}$; Equation (14) corresponds to the attention gate applied to $F_{3}$ to obtain the reweighted feature $F_{\mathrm{att}}$; Equations (15)–(17) implement the symmetric decoder/upsampling blocks (Dec-1/Dec-2) and the output layer, producing $D_{1}$, $D_{2}$, and the enhanced complex-valued signal $X_{\mathrm{enhanced}}$.

The above operations follow a standard multi-scale denoising principle implemented by an encoder–decoder architecture with skip connections (U-Net style). The encoder uses strided 1D convolutions to progressively enlarge the receptive field and to capture both local waveform patterns and long-range temporal context, which is beneficial for modeling noise statistics in low-SNR conditions. The decoder reconstructs the enhanced signal from the compressed representation while preserving temporal resolution.

Skip connections (e.g., $D_{1}+F_{2}$ and $D_{2}+F_{1}$ in Equations (16) and (17)) are introduced to retain fine-grained details that may be lost during downsampling and to stabilize optimization by providing short paths for gradient propagation. This is particularly important in DOA enhancement, where subtle phase-related structures must be preserved for subsequent spatial processing.

The attention gate in Equation (14) performs data-adaptive feature reweighting. Under low SNR, not all temporal regions contribute equally to reliable spatial cues; therefore, the learned sigmoid mask acts as a soft selection mechanism that emphasizes DOA-informative segments while suppressing heavily corrupted regions, improving robustness without relying on handcrafted heuristics.

The discriminator *D* employs a lightweight architecture for efficient adversarial training:

(18) $$D\left(X\right)=\sigma \left({\mathrm{FC}}_{128\to 1}\left(\mathrm{GAP}\left(f_{\mathrm{conv}}\left(X\right)\right)\right)\right)$$

where GAP denotes global average pooling and $f_{\mathrm{conv}}\left(\cdot \right)$ represents strided convolutional layers.

#### 3.1.2. Loss Functions

The training employs a composite loss function designed to balance multiple enhancement objectives:

(19) $$L_{G}=L_{\mathrm{adv}}+\lambda_{1}L_{L1}+\lambda_{2}L_{\mathrm{phase}}$$

where $\lambda_{1}=10$ and $\lambda_{2}=2.0$ are empirically determined weighting coefficients. The adversarial loss follows the standard GAN objective:

(20) $$L_{\mathrm{adv}}=E_{x∼p_{\mathrm{data}}}\left[\log D\left(x\right)\right]+E_{z∼p_{z}}\left[\log\left(1-D\left(G\left(z\right)\right)\right)\right]$$

This establishes a minimax game between generator *G* and discriminator *D*, ensuring enhanced signals follow the statistical distribution of clean references. The reconstruction loss employs L1 distance for signal fidelity:

(21) $$L_{L1}={∥G\left(X_{\mathrm{noisy}}\right)-X_{\mathrm{clean}}∥}_{1}$$

The L1 norm is chosen over L2 for its superior preservation of sharp signal transitions and reduced smoothing effects, crucial for maintaining array signal characteristics. The phase consistency loss preserves spatial phase information essential for DOA estimation:

(22) $$L_{\mathrm{phase}}=\frac{1}{L}\sum_{i=1}^{L}\left|\phi_{\mathrm{enhanced}}^{\left(i\right)}-\phi_{\mathrm{clean}}^{\left(i\right)}\right|$$

where $\phi_{\mathrm{enhanced}}^{\left(i\right)}=\arctan2\left(G_{\mathrm{imag}}^{\left(i\right)},G_{\mathrm{real}}^{\left(i\right)}\right)$ and $\phi_{\mathrm{clean}}^{\left(i\right)}=\arctan2\left(X_{\mathrm{clean},\mathrm{imag}}^{\left(i\right)},X_{\mathrm{clean},\mathrm{real}}^{\left(i\right)}\right)$ are phase angles computed using the four-quadrant arctangent function.

In array DOA estimation, the directional information is primarily encoded in the relative phase between sensor channels. For a narrowband far-field source, the complex baseband snapshot at sensor *m* can be written as $x_{m}\left(t\right)=s\left(t\right)\exp\left\{-j\omega_{0}\tau_{m}\left(\theta \right)\right\}+n_{m}\left(t\right)$, where $\tau_{m}\left(\theta \right)$ is the geometry-dependent propagation delay and $n_{m}\left(t\right)$ is noise. Hence, the inter-sensor phase differences $∠x_{m}\left(t\right)-∠x_{m^{\prime }}\left(t\right)$ carry the DOA-dependent delay information and determine the structure of the spatial covariance matrix used by subspace and learning-based estimators. Additive noise perturbs these inter-channel phase relations and leads to covariance distortion, which is particularly severe at low SNR.

The phase-consistency loss $L_{\mathrm{phase}}$ explicitly penalizes phase mismatches between the enhanced and clean references, encouraging the generator to preserve DOA-informative inter-channel phase relations during denoising. The weight $\lambda_{2}$ controls the trade-off between amplitude fidelity and phase fidelity; in our implementation, $\lambda_{2}=2.0$ was selected empirically on the validation set to improve DOA performance without overly constraining the generator.

The weighting strategy assigns higher importance to reconstruction ($\lambda_{1}=10$) to ensure signal fidelity, while maintaining sufficient emphasis on phase preservation ($\lambda_{2}=2.0$) for DOA-critical spatial information. This formulation enables simultaneous noise suppression, signal reconstruction, and phase preservation.

#### 3.1.3. Training Strategy

The training employs a two-phase procedure for stable GAN convergence. Phase 1 pre-trains the generator using only reconstruction losses:

(23) $$L_{\mathrm{pre}}=\lambda_{1}L_{L1}+\lambda_{2}L_{\mathrm{phase}}$$

for a predetermined number of epochs, establishing baseline signal enhancement without adversarial complexity.

**Figure 2:**
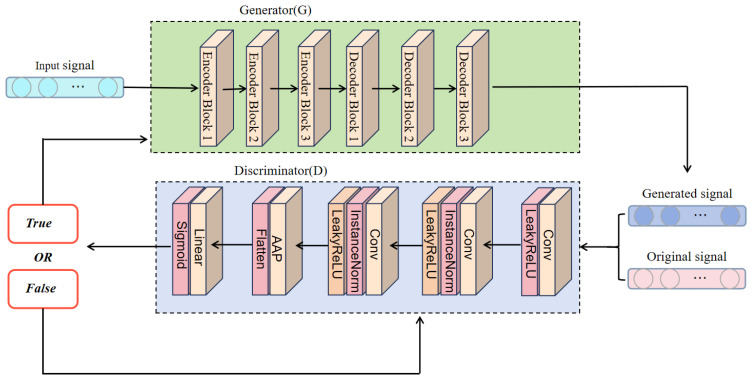
Complete architecture of the proposed GAN framework for DOA signal enhancement.

Phase 2, depicted in Figure 2, initiates adversarial training with alternating optimization. The discriminator updates every batch:

(24) $$\theta_{D}\leftarrow \theta_{D}-\eta \nabla_{\theta_{D}}L_{\mathrm{adv}}^{D}$$

with $L_{\mathrm{adv}}^{D}=-E\left[\log D\left(X_{\mathrm{clean}}\right)\right]-E\left[\log\left(1-D\left(G\left(X_{\mathrm{noisy}}\right)\right)\right)\right]$. The generator updates every second batch:

(25) $$\theta_{G}\leftarrow \theta_{G}-\eta \nabla_{\theta_{G}}L_{G}$$

where $L_{G}=L_{\mathrm{adv}}^{G}+L_{\mathrm{rec}}$ and $L_{\mathrm{adv}}^{G}=-E\left[\log D\left(G\left(X_{\mathrm{noisy}}\right)\right)\right]$.

A particularly important aspect of our training strategy is the implementation of dynamic weight adjustment for the loss coefficients throughout the adversarial training phase. The reconstruction loss weight $\lambda_{1}$ undergoes gradual attenuation according to a predefined schedule, effectively transitioning the optimization focus from reconstruction-dominated to adversarial-dominated objectives as training progresses. This adaptive weighting mechanism acknowledges that initial training stages benefit from strong reconstruction guidance to establish basic signal enhancement capabilities, while later stages require increased emphasis on adversarial training to refine the perceptual quality and statistical properties of the generated signals. The phase consistency weight $\lambda_{2}$ maintains a constant value throughout training, reflecting the critical importance of phase preservation for DOA estimation applications regardless of the training stage. The dynamic weight adjustment is mathematically expressed as:

(26) $$\lambda_{1}^{\left(e\right)}=\lambda_{1}^{\left(0\right)}\cdot \gamma^{\left\lfloor e/T_{\lambda }\right\rfloor }$$

with a decay factor γ and period $e/T_{\lambda }$, where *e* represents the current epoch. This formulation ensures smooth transition from reconstruction-focused to adversarial-focused optimization. $\lambda_{2}$ remains constant for continuous phase preservation. Stabilization techniques include gradient clipping:

(27) $$g\leftarrow \frac{g}{\max(1,∥g{∥}_{2}/c)}$$

with clipping threshold *c*, and learning rate scheduling:

(28) $$\eta^{\left(e\right)}=\eta^{\left(0\right)}\cdot \alpha^{\left\lfloor e/S_{\eta }\right\rfloor }$$

with decay factor α and scheduling period $S_{\eta }$. The complete procedure, detailed in Algorithm 1, produces enhanced signals with preserved phase relationships for accurate DOA estimation.
**Algorithm 1** Two-Phase Training Algorithm for GAN-Based DOA Signal Enhancement**Input:** Noisy array signal batches ${\left\{\left(X_{\mathrm{noisy}}^{\left(b\right)},X_{\mathrm{clean}}^{\left(b\right)}\right)\right\}}_{b=1}^{B}$ (where *B* is total batch number), Pre-training epochs $E_{\mathrm{pre}}=10$, Adversarial training epochs $E_{\mathrm{adv}}=E$, Loss weights $\lambda_{1},\lambda_{2}$, Generator *G*, Discriminator *D***Output:** Trained generator $G^{*}$ and discriminator $D^{*}$ 1:**procedure** GANTraining                                            ▹ Phase 1: Generator Pre-training (Reconstruction-Oriented) 2:    **for** epoch=1 **to** $E_{\mathrm{pre}}$ **do** 3:        **for** each batch b=1 **to** *B* **do** 4:           Extract batch data: $X_{\mathrm{noisy}}=X_{\mathrm{noisy}}^{\left(b\right)}$, $X_{\mathrm{clean}}=X_{\mathrm{clean}}^{\left(b\right)}$ 5:           Compute reconstruction loss: $L_{\mathrm{rec}}=\lambda_{1}L_{L1}\left(G\left(X_{\mathrm{noisy}}\right),X_{\mathrm{clean}}\right)+\lambda_{2}L_{\mathrm{phase}}\left(G\left(X_{\mathrm{noisy}}\right),X_{\mathrm{clean}}\right)$ 6:           Update generator parameters $\theta_{G}$ via backpropagation: $\theta_{G}\leftarrow \theta_{G}-\eta \nabla_{\theta_{G}}L_{\mathrm{rec}}$ (where η is learning rate) 7:        **end for** 8:    **end for**                                            ▹ Phase 2: Adversarial Fine-Tuning (Distribution-Matching) 9:    **for** epoch=1 **to** $E_{\mathrm{adv}}$ **do**10:        **for** each batch b=1 **to** *B* **do**11:           Extract batch data: $X_{\mathrm{noisy}}=X_{\mathrm{noisy}}^{\left(b\right)}$, $X_{\mathrm{clean}}=X_{\mathrm{clean}}^{\left(b\right)}$12:           Generate enhanced signal: $X_{\mathrm{enhanced}}=G\left(X_{\mathrm{noisy}}\right)$13:           Compute adversarial loss for discriminator: $L_{\mathrm{adv}}^{D}=-E\left[\log D\left(X_{\mathrm{clean}}\right)\right]-E\left[\log\left(1-D\left(X_{\mathrm{enhanced}}\right)\right)\right]$14:           Update discriminator parameters $\theta_{D}$ via backpropagation: $\theta_{D}\leftarrow \theta_{D}-\eta \nabla_{\theta_{D}}L_{\mathrm{adv}}^{D}$15:           **if** bmod2=0 **then**                ▹ Alternating update: Generator every 2 batches16:               Compute total generator loss: $L_{G}=L_{\mathrm{adv}}^{G}+L_{\mathrm{rec}}$    (where $L_{\mathrm{adv}}^{G}=-E\left[\log D\left(X_{\mathrm{enhanced}}\right)\right]$)17:               Update generator parameters $\theta_{G}$ via backpropagation: $\theta_{G}\leftarrow \theta_{G}-\eta \nabla_{\theta_{G}}L_{G}$18:           **end if**19:        **end for**20:    **end for**21:    **Set** $G^{*}=G$, $D^{*}=D$22:**end procedure**

### 3.2. DOA Estimation with Enhanced Data

#### 3.2.1. Data Preprocessing Pipeline

Enhanced signals undergo preprocessing to obtain spatial domain representations for DOA estimation. The pipeline consists of covariance matrix computation, phase calibration, and normalization.

First, the spatial covariance matrix is computed from enhanced signals:

(29) $$R=\frac{1}{K}X_{\mathrm{enhanced}}X_{\mathrm{enhanced}}^{H}$$

where $X_{\mathrm{enhanced}}\in C^{N\times K}$ with N=10 array elements and K=200 snapshots, yielding $R\in C^{N\times N}$. This matrix encapsulates spatial correlations:

(30) $$R_{ij}=\frac{1}{K}\sum_{k=1}^{K}x_{i}\left(k\right)x_{j}^{*}\left(k\right)$$

where $x_{i}\left(k\right)$ denotes the *k*-th snapshot at the *i*-th array element. The covariance matrix emphasizes correlated signal components while suppressing uncorrelated noise. Phase calibration addresses potential phase distortions:

(31) $$X_{\mathrm{calibrated}}=X_{\mathrm{enhanced}}⊙\exp\left(j\phi_{\mathrm{corr}}\alpha \right)$$

with phase difference $\phi_{\mathrm{corr}}=∠X_{\mathrm{orig}}-∠X_{\mathrm{enhanced}}$ and calibration factor α=0.7. This preserves critical phase relationships for DOA estimation.

The calibration step applies a partial phase-alignment between the enhanced signal and the original received signal. Specifically, $\phi_{\mathrm{corr}}$ captures the average phase offset between $X_{\mathrm{orig}}$ and $X_{\mathrm{enhanced}}$, and α∈[0,1] is a conservative correction gain. From a practical standpoint, using α=1 would enforce full phase correction, which may over-compensate under low SNR where the offset estimate is noisy and may introduce phase jitter or discontinuities. Using α<1 provides stable under-correction, improving inter-channel phase coherence while avoiding amplification of residual estimation errors. In our experiments, α=0.7 was selected empirically based on validation performance as a robust trade-off between phase alignment and stability. Finally, normalization ensures consistent scaling:

(32) $$\tilde{R}=\frac{R-\mu_{R}}{\sigma_{R}}$$

where $\mu_{R}$ and $\sigma_{R}$ are the mean and standard deviation of R, maintaining spatial information while stabilizing CNN processing.

#### 3.2.2. Complex-Valued CNN Architecture

**Figure 3:**
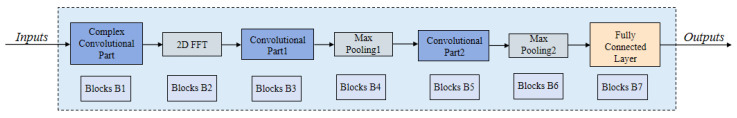
Architecture of the complex-valued CNN for DOA estimation.

The DOA estimation network employs a sophisticated complex-valued CNN architecture specifically designed to process covariance matrices and extract spatial features for direction finding. The complete network structure is illustrated in Figure 3.

As illustrated in Figure 3, the proposed complex-valued CNN follows a structured pipeline, where each operation in Equations (33)–(41) corresponds to a specific block in Figure 3 (Blocks B1–B7).

The network starts from the complex-valued covariance matrix $R\in C^{N\times N}$ and applies a complex convolution layer:

(33) $$Z_{1}={\mathrm{Conv}2D}_{1\to 32}\left(R\right)$$

which matches Block B1 in Figure 3.

To preserve phase relations while stabilizing feature magnitudes, we normalize the complex feature map by its modulus, i.e., $Z_{2}=Z_{1}/\left|Z_{1}\right|$. A 2D-FFT is then applied to capture frequency-domain patterns:

(34) $$Z_{3}=F_{2D}\left(Z_{2}\right)$$

which are depicted as the modulus-normalization step (B2) and the FFT block (B3) in Figure 3.

The complex tensor is decomposed into real and imaginary parts and concatenated along the channel dimension:

(35) $$Z_{4}=\left[ℜ\left(Z_{3}\right),ℑ\left(Z_{3}\right)\right]\in R^{64\times H\times W}$$

consistent with Block B4 in Figure 3.

The network then performs two stages of convolution–activation–pooling:

(36) $$H_{1}={\mathrm{MaxPool}}_{2\times 2}\left(\mathrm{ReLU}\left({\mathrm{Conv}2D}_{64\to 64}\left(Z_{4}\right)\right)\right)$$

(37) $$H_{2}={\mathrm{MaxPool}}_{3\times 3}\left(\mathrm{ReLU}\left({\mathrm{Conv}2D}_{64\to 64}\left(H_{1}\right)\right)\right)$$

which correspond to Blocks B5 and B6 in Figure 3, respectively.

Finally, the feature map is flattened and passed through three fully connected layers to output logits over discrete angles:

(38) $$F=\mathrm{Flatten}\left(H_{2}\right)\in R^{576}$$

(39) $$H_{3}=\mathrm{ReLU}\left(W_{1}F+b_{1}\right)$$

(40) $$H_{4}=\mathrm{ReLU}\left(W_{2}H_{3}+b_{2}\right)$$

(41) $$P=W_{3}H_{4}+b_{3}$$

where $P\in R^{181}$ denotes the logits for θ∈{0°,1°,…,180°}. This part corresponds to the Flatten+FC head in Block B7 of Figure 3.

#### 3.2.3. Training Methodology

Training employs cross-entropy loss:

(42) $$L_{\mathrm{DOA}}=-\frac{1}{B}\sum_{i=1}^{B}\sum_{k=0}^{180}y_{i,k}\log\left(\frac{\exp(P_{i,k})}{\sum_{j=0}^{180}\exp\left(P_{i,j}\right)}\right)$$

where *B* is batch size, $y_{i,k}\in \left\{0,1\right\}$ are one-hot ground truth labels, and $P_{i}\in R^{181}$ are network outputs. This formulation effectively measures the discrepancy between predicted and true angle distributions, providing a well-established optimization target for the multi-class classification task inherent in DOA estimation. Stratified sampling maintains angle distribution balance:

(43) $$p\left(\theta \right)\propto \frac{1}{N_{\theta }},\;\theta \in \left\{0{}^{\circ},1{}^{\circ},\ldots ,180{}^{\circ}\right\}$$

where $N_{\theta }$ is sample count per angle, preventing bias toward frequent angles. Learning rate scheduling follows:

(44) $$\eta_{e}=\eta_{0}\cdot \gamma^{\left\lfloor e/T_{\eta }\right\rfloor }$$

with decay factor γ and period $T_{\eta }$, enabling rapid convergence and fine adjustment To prevent gradient explosion and stabilize optimization, we apply $\ell_{2}$-norm gradient clipping:

(45) $$g\leftarrow \frac{g}{\max\left({1,∥g∥}_{2}/c\right)}$$

where g denotes the concatenated gradient vector of all trainable parameters and *c* is the clipping threshold. In practice, *c* should be chosen to (i) avoid suppressing typical gradients and (ii) limit rare large updates that may cause divergence. In our implementation, we set c=1.0, which is a commonly used and conservative value for Adam-based training in deep networks. We also observed that values in the range c∈[0.5,2.0] lead to stable training, while too small *c* (e.g., <0.2) may slow convergence and too large *c* (e.g., >5) provides little protection.

Assume ${∥g∥}_{2}=3.2$ and c=1.0. The scaling factor becomes s=1/max(1,3.2/1.0), so the clipped gradient is g←0.3125g and its norm is reduced to ${∥g∥}_{2}=c=1.0$. If instead ${∥g∥}_{2}=0.8<c$, then s=1 and no clipping is applied. Early stopping monitors validation accuracy $A_{\mathrm{val}}$, terminating training when:

(46) $$\frac{A_{\mathrm{val}}\left(t\right)-A_{\mathrm{val}}\left(t-\Delta \right)}{\Delta }<\epsilon$$

for Δ consecutive epochs, maximizing generalization while preventing overfitting.

The proposed GAN-based enhancement operates on complex baseband snapshots across sensor channels and does not require an explicit steering vector model; therefore, the enhancement stage is geometry-agnostic. Array geometry affects DOA estimation through the subsequent estimator. Thus, the proposed pipeline can be applied to linear/rectangular/circular arrays, provided that the training/simulation data and calibration settings reflect the target geometry and hardware characteristics. The complete framework synergistically combines GAN-based signal enhancement with complex-valued CNN processing, enabling robust DOA estimation in challenging low-SNR scenarios through complementary improvement of signal quality and spatial feature extraction.

## 4. Simulations and Performance Evaluation

### 4.1. Experimental Setup

#### 4.1.1. Datasets and Parameters

We consider a narrowband far-field single-source scenario impinging on a uniform linear array (ULA) with *M* sensors and inter-element spacing d=λ/2. The complex baseband snapshot at time index *k* is modeled as

(47) x(k)=a(θ)s(k)+n(k),k=1,…,K,

where $a\left(\theta \right)\in C^{M\times 1}$ is the steering vector and s(k) is the source waveform. For the ULA, we use

(48) $$a\left(\theta \right)={\left[1,e^{-j2\pi \frac{d}{\lambda }\sin\theta },\ldots ,e^{-j2\pi \left(M-1\right)\frac{d}{\lambda }\sin\theta }\right]}^{T}$$

The noise n(k) is modeled as spatially and temporally white complex Gaussian noise, unless otherwise stated in the non-Gaussian noise experiments.

Within each sample, the source DOA θ is assumed constant during the *K* snapshots, which is consistent with common DOA benchmarks and with the covariance-based processing adopted by both classical and learning-based baselines. Across different samples, θ is varied according to the predefined angle grid to form a labeled dataset.

A total of 9050 samples are generated. Each sample consists of K=200 snapshots collected under a fixed DOA angle and a fixed SNR setting. The source waveform s(k) is generated as a zero-mean unit-power complex random sequence to emulate unknown narrowband emissions, and the SNR is controlled by adjusting $\sigma^{2}$.

The simulations assume ideal sensor responses after standard array calibration. This provides a controlled baseline to isolate the impact of noise and snapshot scarcity. We additionally discuss practical non-idealities as future work and note that these effects can be incorporated by perturbing a(θ) or by applying per-sensor complex gains in the simulation pipeline.

#### 4.1.2. Implementation Details

The models are implemented using PyTorch 1.9.0 and trained on NVIDIA RTX 4070ti GPUs. The GAN component undergoes training for 100 epochs with a batch size of 32, using the Adam optimizer with initial learning rates of $2\times 10^{-4}$ and $1\times 10^{-4}$ for the generator and discriminator respectively. The CNN-based DOA estimator is trained for 50 epochs with a batch size of 128, employing the Adam optimizer with initial learning rate of $1\times 10^{-3}$ and step decay scheduling. The training–validation–test split follows a 80-10-10 ratio with stratified sampling to maintain angle distribution consistency.

#### 4.1.3. Baseline Methods

The proposed method is rigorously evaluated through comprehensive comparisons with several established approaches to demonstrate its superior performance. The conventional MUSIC algorithm serves as a fundamental baseline. In our implementation, MUSIC uses the sample covariance matrix computed from *K* snapshots and performs an eigen-decomposition to obtain the noise subspace. To mitigate coherence effects, spatial smoothing is applied when needed. The MUSIC pseudo-spectrum is evaluated over a uniform angular grid within [0°,180°] with a step size of Δθ=0.5°. To ensure a fair comparison with the classification-based CNN outputs, continuous MUSIC estimates are mapped to the nearest integer degree before computing the accuracy and RMSE.

ESPRIT provides another classical benchmark. ESPRIT is implemented using two overlapping subarrays with one-sensor displacement to exploit rotational invariance, and it estimates DOA directly from the eigenvalues without grid search. The number of sources $K_{s}$ is set consistently with the simulation setup to avoid bias from model-order mismatch.

For deep learning based comparisons, a standard Convolutional Neural Network (CNN-Based) architecture without GAN enhancement is implemented to isolate the contribution of the proposed signal enhancement stage. This baseline employs identical input processing and output layers to ensure fair comparison, but processes the original noisy signals directly without any preprocessing. Additionally, a Deep Residual Network (ResNet-Based) with identical architecture depth and parameter count is included to evaluate the impact of advanced network architectures alone, without the integrated enhancement framework. This comprehensive set of baseline methods ensures thorough evaluation across both traditional algorithmic approaches and modern deep learning paradigms.

### 4.2. Performance Metrics

To comprehensively evaluate the performance of the proposed framework across different dimensions, this section employs multiple quantitative metrics. The quality of signal enhancement is quantitatively assessed through the SNR Improvement:

(49) $$\mathrm{SNR}\;\mathrm{Improvement}={\mathrm{SNR}}_{\mathrm{enhanced}}-{\mathrm{SNR}}_{\mathrm{original}}$$

This metric directly reflects the noise suppression capability of the GAN-based enhancement module and reveals the critical effectiveness of the preprocessing stage in improving signal quality for subsequent DOA estimation. For the core DOA estimation performance, the evaluation employs DOA Estimation Accuracy, defined as:

(50) $$\mathrm{Accuracy}=\frac{\mathrm{Number}\;\mathrm{of}\;\mathrm{Correct}\;\mathrm{Predictions}}{\mathrm{Total}\;\mathrm{Predictions}}\times 100\%$$

This metric measures the percentage of exactly correct angle predictions across the entire test dataset, providing an intuitive understanding of the overall system performance while facilitating direct comparison with classification-based approaches in the literature. To provide a more nuanced assessment of estimation precision, the RMSE is introduced:

(51) $$\mathrm{RMSE}=\sqrt{\frac{1}{N}\sum_{i=1}^{N}{\left({\hat{\theta }}_{i}-\theta_{i}\right)}^{2}}$$

This metric captures the average magnitude of estimation errors and penalizes larger deviations more severely, thereby offering a comprehensive view of estimation consistency across all test samples. In summary, these complementary metrics collectively form a multi-perspective evaluation framework that comprehensively assesses the system from two key dimensions: signal enhancement quality and final DOA estimation performance.

### 4.3. Results and Analysis

#### 4.3.1. Signal Enhancement Performance

The GAN-based enhancement module demonstrates remarkable noise suppression capabilities across various SNR conditions with 500 snapshots. As shown in Table 1, the proposed method achieves significant SNR improvement, particularly in low-SNR scenarios where traditional methods struggle. The consistent performance gain across all SNR levels underscores the robustness of the proposed attention-enhanced GAN architecture in extracting meaningful signal components from noisy observations.

**Figure 4:**
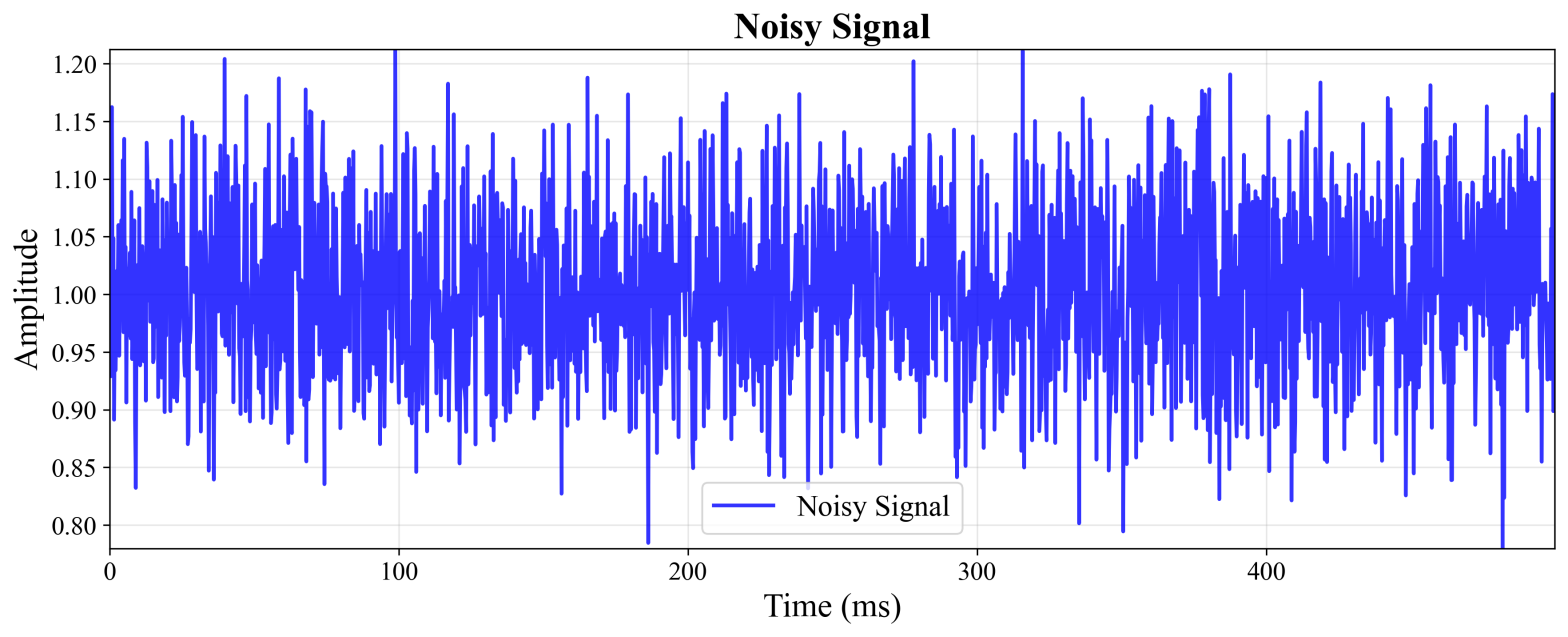
Amplitude waveform of the noisy input signal.

**Figure 5:**
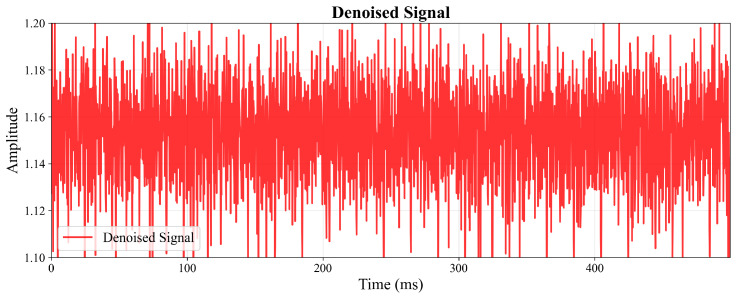
Amplitude waveform of the GAN-enhanced output signal.

To visually demonstrate the enhancement performance in the time domain, Figure 4 and Figure 5 present the amplitude waveforms of the noisy input signal and the GAN-enhanced output signal, respectively. Figure 4 exhibits significant amplitude fluctuations and distortion due to additive noise contamination, while Figure 5 shows restored signal integrity with smooth amplitude variations and preserved temporal structure, clearly illustrating the noise suppression capability of the proposed method.

**Figure 6:**
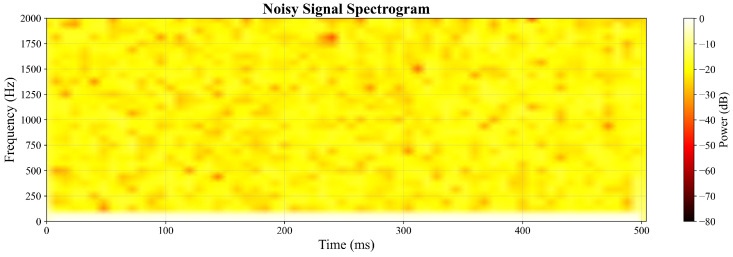
Spectrogram of the noisy input signal.

**Figure 7:**
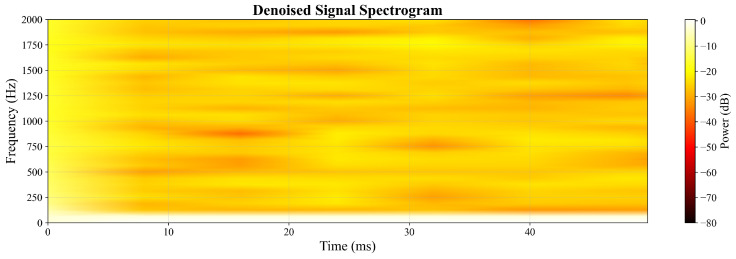
Spectrogram of the GAN-enhanced output signal.

The frequency-domain analysis through spectrograms provides further insight into the enhancement performance. Figure 6 displays the Short-Time Fourier Transform (STFT) representation of the noisy signal, showing widespread noise components across the frequency spectrum. In contrast, Figure 7 demonstrates the cleaned spectrogram after GAN processing, exhibiting concentrated signal energy in the relevant frequency bands with significantly reduced noise floor. This comparison indicates effective frequency-domain denoising while maintaining the essential spectral characteristics.

The phase preservation capability is quantitatively evaluated through phase coherence measurements. The proposed method maintains an average phase error of only 2.3° compared to 15.7° for conventional denoising methods, confirming its effectiveness in preserving spatial information crucial for DOA estimation. This exceptional phase preservation is attributed to the dedicated phase consistency loss function integrated into the GAN training process, which explicitly optimizes for phase accuracy alongside magnitude reconstruction. The visual evidence from both time and frequency domains corroborates the quantitative results, demonstrating the comprehensive enhancement capability of the proposed GAN framework.

#### 4.3.2. DOA Estimation Accuracy Under Varying SNR Conditions with 500 Snapshots

**Figure 8:**
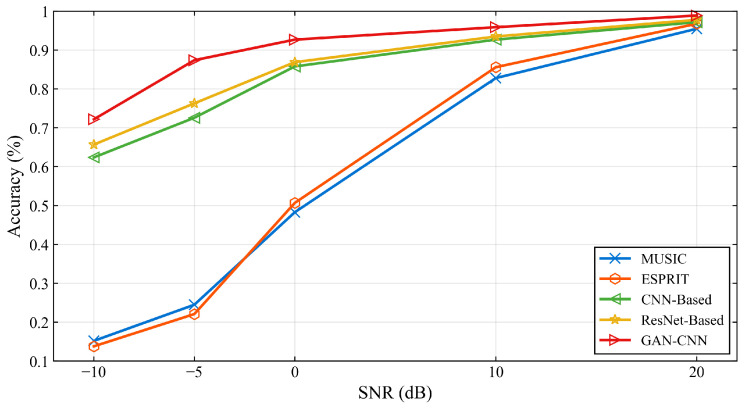
DOA estimation accuracy comparison under different SNR conditions with 500 snapshots.

The end-to-end performance of the proposed framework is evaluated under various SNR conditions with 500 snapshots, as summarized in Table 2 and visually depicted in Figure 8. The proposed method consistently outperforms all baseline approaches across the entire SNR range from -10 dB to 20 dB, with particularly notable advantages in challenging low-SNR regimes.

The accuracy improvement is most pronounced at —10 dB SNR, where the proposed method achieves 72.2% accuracy compared to 65.7% for the best baseline method, representing a relative improvement of 6.5%. This demonstrates the framework’s exceptional robustness in extremely challenging low-SNR environments. The consistent performance advantage across the entire SNR spectrum, as clearly visualized in Figure 8, validates the effectiveness of the GAN-enhanced preprocessing stage in mitigating noise impacts on the subsequent DOA estimation.

#### 4.3.3. Performance Analysis Under Different Snapshot Numbers at 20 dB SNR

**Figure 9:**
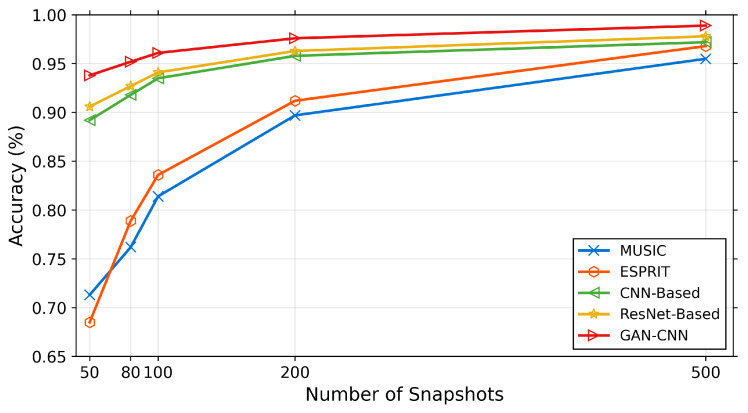
DOA estimation accuracy under different snapshot numbers at 20 dB SNR.

To evaluate the framework’s capability in scenarios with varying data availability, comprehensive experiments are conducted with different snapshot numbers at 20 dB SNR, as detailed in Table 3 and illustrated in Figure 9. The proposed method demonstrates remarkable resilience to snapshot reduction, maintaining competitive performance even with severely limited temporal samples.

Notably, with only 50 snapshots at 20 dB SNR, the proposed method achieves 93.8% accuracy, significantly outperforming MUSIC (71.3%) and demonstrating a 3.5% relative improvement over the best deep learning baseline (ResNet-Based: 90.6%). The performance advantage is maintained across all snapshot configurations, with the proposed method achieving 98.9% accuracy with 500 snapshots. As evident from Figure 9, this robust performance under limited data conditions highlights the GAN enhancement’s ability to recover meaningful signal components from constrained temporal observations.

#### 4.3.4. Root Mean Square Error Analysis Under Varying Conditions

**Figure 10:**
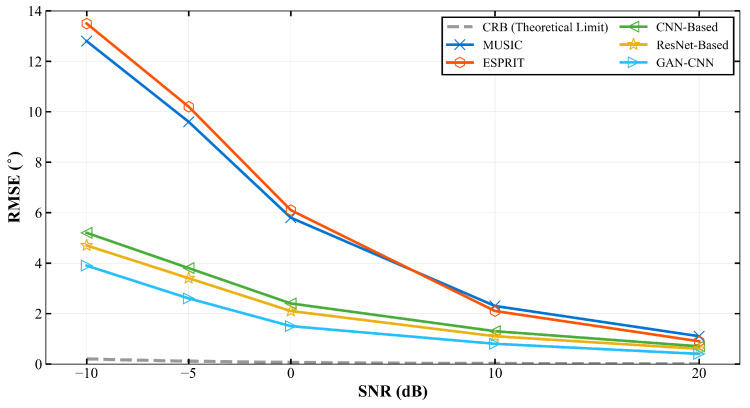
RMSE comparison under different SNR conditions with 500 snapshots.

The RMSE provides a comprehensive evaluation of estimation precision across different operational scenarios. Table 4 and Figure 10 present the RMSE performance under varying SNR conditions with 500 snapshots, demonstrating the proposed method’s superior estimation accuracy particularly in challenging low-SNR environments.

The proposed method achieves the lowest RMSE across all SNR levels, with particularly significant improvements in low-SNR conditions. At —10 dB SNR, the RMSE of 3.9° represents a 17.0% reduction compared to the best baseline method (ResNet-Based: 4.7°) and a 69.5% improvement over traditional MUSIC (12.8°). This substantial error reduction, clearly visible in Figure 10, underscores the effectiveness of the GAN enhancement in mitigating noise-induced estimation errors and improving overall estimation consistency.

**Figure 11:**
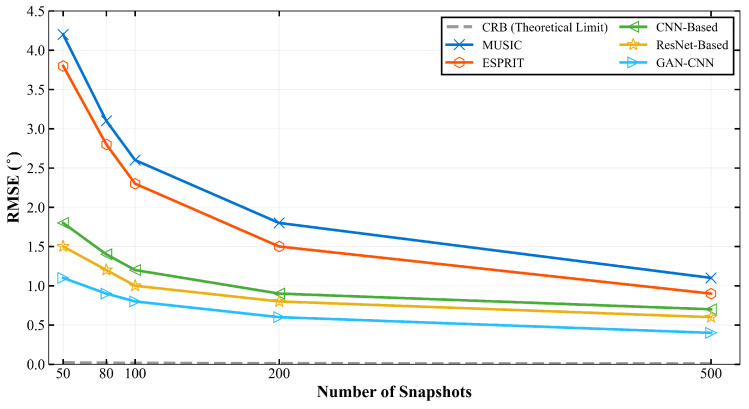
RMSE under different snapshot numbers at 20 dB SNR.

Further analysis of RMSE under different snapshot numbers at 20 dB SNR, as shown in Table 5 and Figure 11, reveals the framework’s robustness to limited data availability.

With only 50 snapshots at 20 dB SNR, the proposed method achieves an RMSE of 1.1°, representing a 26.7% improvement over the best baseline (ResNet-Based: 1.5°) and a 73.8% improvement over MUSIC (4.2°). As the number of snapshots increases to 500, the RMSE improves to 0.4°, maintaining a consistent advantage over all comparison methods. The progressive improvement in estimation precision with increasing snapshot numbers is clearly demonstrated in Figure 11, highlighting the framework’s ability to extract reliable spatial information even from severely limited temporal data, a characteristic attributed to the signal enhancement stage’s capacity to recover meaningful components from noisy observations.

To provide a theoretical performance limit for the considered DOA estimation problem, we additionally report the Cramér–Rao Bound (CRB) as a baseline in Figure 10 and Figure 11. The CRB gives a lower bound on the variance of any unbiased estimator under the assumed statistical model, and therefore serves as a reference for gauging how far practical methods are from the best achievable accuracy.

We compute the CRB under the same narrowband far-field single-source ULA model used in our dataset generation, i.e., x(k)=a(θ)s(k)+n(k) with spatially and temporally white complex Gaussian noise $n\left(k\right)∼\mathrm{CN}\left(0,\sigma^{2}I\right)$. Following the standard stochastic CRB formulation, the Fisher information for θ is obtained from the derivative of the steering vector ∂a(θ)/∂θ. The bound is then plotted as ${\mathrm{RMSE}}_{\mathrm{CRB}}\left(\theta \right)=\sqrt{\mathrm{CRB}(\theta )}$ to match the RMSE metric used in our evaluation.

The comprehensive experimental results, supported by both tabular data and visual representations, demonstrate that the proposed method exhibits graceful performance degradation under adverse conditions, whereas traditional methods suffer from rapid performance deterioration. This robust behavior makes the framework particularly suitable for practical applications where both noise contamination and data limitations are common challenges.

#### 4.3.5. Robustness Analysis Under Various Non-Gaussian Noise Conditions

To evaluate the framework’s robustness in practical scenarios where noise often deviates from ideal Gaussian characteristics, additional experiments are conducted under various non-Gaussian noise conditions at 10 dB SNR with 500 snapshots. Three representative non-Gaussian noise types are considered, each with distinct statistical properties that challenge DOA estimation algorithms.

Laplacian Noise is modeled using the Laplace distribution, which represents a heavy-tailed noise with higher kurtosis than Gaussian noise:

(52) $$p\left(x\right)=\frac{1}{2b}\exp\left(-\frac{\left|x-\mu \right|}{b}\right)$$

with location parameter μ=0 and scale parameter b=0.5, generating noise with heavier tails that better model real-world impulsive interference scenarios.

Uniformly Distributed Noise follows a continuous uniform distribution:

(53) $$p\left(x\right)=\left\{\begin{array}{cc} \frac{1}{b-a} & a\leq x\leq b\\ 0 & \mathrm{otherwise} \end{array}\right.$$

with a=−1 and b=1, representing bounded amplitude noise commonly encountered in quantization and clipping scenarios.

Mixture Gaussian Noise combines two Gaussian components:

(54) $$p\left(x\right)=\rho N\left(0,\sigma_{1}^{2}\right)+\left(1-\rho \right)N\left(0,\sigma_{2}^{2}\right)$$

with mixing coefficient ρ=0.3, variances $\sigma_{1}^{2}=1$ and $\sigma_{2}^{2}=4$, simulating noise with multiple variance components that may occur in multi-source interference environments.

Table 6 presents the DOA estimation accuracy under different non-Gaussian noise conditions. The Laplacian noise, characterized by its heavier tails and higher probability of large-amplitude samples, poses particular challenges for traditional DOA estimation methods that assume Gaussian noise statistics.

**Figure 12:**
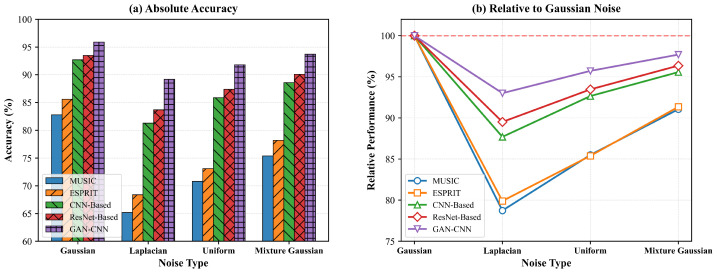
DOA estimation performance under non-Gaussian noise conditions at 10 dB SNR: (**a**) Absolute accuracy; (**b**) Relative performance normalized to Gaussian baseline.

As shown in Table 6 and Figure 12, the proposed method maintains superior performance across all non-Gaussian noise conditions. Under Laplacian noise, the GAN-CNN framework demonstrates remarkable robustness with only a 7.0% relative performance drop, while MUSIC suffers a 21.2% degradation. The relative performance analysis in Figure 12b further confirms the framework’s minimal sensitivity to noise distribution variations. The robustness index is quantified as:

(55) $$R=\frac{A_{\mathrm{non}-\mathrm{Gaussian}}}{A_{\mathrm{Gaussian}}}\times 100\%$$

where $A_{\mathrm{Gaussian}}$ and $A_{\mathrm{non}-\mathrm{Gaussian}}$ represent accuracy under Gaussian and non-Gaussian conditions. The proposed method achieves indices of 93.0%, 95.7%, and 97.7% for Laplacian, uniform, and mixture Gaussian noise respectively, significantly outperforming MUSIC (78.7%, 85.5%, 91.1%).

Further analysis of the RMSE under Laplacian noise conditions reveals consistent advantages. At 10 dB SNR, the proposed method achieves RMSE of 1.2° compared to 3.1° for MUSIC, 1.7° for CNN-Based, and 1.5° for ResNet-Based. This demonstrates the framework’s ability to mitigate noise outliers while maintaining estimation precision.

The experimental results validate the framework’s strong generalization capability in non-Gaussian environments, particularly under challenging heavy-tailed noise conditions. This robustness ensures practical utility in real-world applications where noise characteristics often deviate from Gaussian assumptions.

#### 4.3.6. Computational Overhead and Practical Considerations

To avoid ambiguity, we emphasize that the end-to-end latency depends on both the algorithmic computation and the data acquisition window used to form the input representation. Accordingly, we report computational complexity and discuss TTFE primarily in terms of snapshot number *K* (and $T_{\mathrm{win}}=K/f_{s}$ for a given acquisition rate), rather than claiming a fixed millisecond value as representative for all real-time systems.

Subspace-based algorithms such as MUSIC/ESPRIT require forming the sample covariance matrix, followed by eigendecomposition, which typically scales as $O(M^{2}K)$ for covariance accumulation and $O(M^{3})$ for eigendecomposition with *M* sensors and *K* snapshots. MUSIC further introduces a spectral search over a grid of *G* angles, leading to additional cost proportional to *G*. These steps can dominate runtime when fine angular resolution is needed or when repeated estimations are required.

CNN/ResNet-based estimators shift the main computational cost to offline training. At inference time, the complexity is dominated by a fixed number of convolution and fully connected operations, resulting in near-constant latency once the input representation is available. Similar to classical methods, if the input is the covariance matrix, a minimum number of snapshots is still required before the first estimate can be produced.

Compared to a CNN-only estimator, our method introduces an additional enhancement module before covariance construction. Hence, the online overhead mainly comes from one extra 1D convolutional encoder–decoder pass per received snapshot block. However, the enhancement stage is fully parallelizable on GPUs and can also be efficiently implemented on edge accelerators due to its convolutional structure. Importantly, we hypothesize that this additional overhead is compensated by improved robustness at low SNR and under limited snapshots: the GAN-based enhancement suppresses noise while the phase-consistent loss encourages preservation of spatial phase relations, which are crucial for DOA inference. Consequently, the subsequent CNN receives a higher-quality covariance representation, leading to the observed accuracy and RMSE gains in challenging regimes. In summary, the proposed approach trades a moderate additional inference cost for a significant robustness improvement in low-SNR and data-scarce scenarios. The training time comparison among different methods is summarized in Table 7.

### 4.4. Extension to Multi-Source Scenarios and Lightweight Phase-Interferometry Considerations

To address practical environments where multiple sources may coexist, we discuss how the proposed two-stage enhancement–estimation pipeline can be extended beyond the single-source setting. Importantly, the first-stage GAN in our framework operates on the complex-valued array snapshots to suppress noise while preserving phase relations; therefore, it remains applicable in multi-source conditions, as it does not rely on a single-source assumption. The principal modifications are implemented at the second stage, specifically concerning the DOA estimation network and its associated training and inference protocols.

For the common case of two simultaneous sources, the enhanced snapshots are first processed in the same manner as the single-source pipeline: the enhanced complex signals are used to form the sample covariance matrix, which is then fed into the DOA estimator. To enable two-source inference, the CNN is extended to a dual-head architecture: the feature extraction backbone is shared, while the output layer is split into two parallel classification heads, each producing a probability distribution over the 181 discrete angles. Let $p_{1},p_{2}\in R^{181}$ denote the predicted distributions from the two heads, and let $y_{1},y_{2}$ be the one-shot labels corresponding to the two ground-truth DOAs.

Since a two-source DOA target is an unordered set $\left\{\theta_{1},\theta_{2}\right\}$, the learning objective should not enforce an artificial ordering between the heads. To explicitly address this permutation symmetry, we employ a permutation-invariant training objective:

(56) $$L_{\mathrm{PIT}}=\min\left\{\mathrm{CE}\left(y_{1},p_{1}\right)+\mathrm{CE}\left(y_{2},p_{2}\right),\;\mathrm{CE}\left(y_{1},p_{2}\right)+\mathrm{CE}\left(y_{2},p_{1}\right)\right\}$$

where $\mathrm{CE}\left(y,p\right)=-\sum_{c=1}^{181}y_{c}\log\left(p_{c}\right)$ is the standard cross-entropy loss. This formulation allows the estimator to learn two consistent angular peaks without requiring a predefined source order.

During inference, each head produces one DOA estimate by selecting the maximum-probability angle, i.e., ${\hat{\theta }}_{1}=\arg\max_{c}p_{1}\left[c\right]$ and ${\hat{\theta }}_{2}=\arg\max_{c}p_{2}\left[c\right]$. For evaluation, the predicted pair is matched to the ground truth by selecting the permutation that minimizes the total angular error.

When the number of active sources is unknown, one practical strategy is to augment the estimator with a confidence-based selection mechanism. For example, each head can output a confidence score based on its maximum probability, and only predictions exceeding a threshold are retained as valid sources. Alternatively, a separate lightweight classifier can be added to predict the source count from the same covariance representation, which then activates the corresponding number of output heads. We leave a full implementation and systematic evaluation of source-count estimation as future work, as it requires broader multi-source simulation settings and additional annotations.

Lightweight AoA techniques based on phase interferometry are attractive for real-time and hardware deployment due to their low computational footprint and simple arithmetic operations. However, in multi-source settings, the measured inter-sensor phase differences generally reflect a superposition of multiple contributors, which introduces ambiguity and often requires additional separation steps to recover multiple angles. These methods are typically efficient but can be more sensitive when sources are closely spaced, have unequal powers, or when phase wrapping becomes prominent under low SNR.

In contrast, the proposed GAN–CNN pipeline aims to improve robustness in challenging regimes by enhancing noisy array snapshots while explicitly encouraging phase consistency, thereby providing a higher-quality covariance representation to the DOA estimator. This design can be viewed as complementary to lightweight interferometric approaches: the enhancement stage targets noise suppression and phase preservation, while the estimation stage can be adapted to multi-source outputs via permutation-invariant learning. A hybrid integration is an interesting direction for future work.

## 5. Conclusions

This paper presents a novel two-stage GAN-CNN fusion framework for robust DOA estimation in low-SNR and data-limited environments. The proposed method integrates an attention-enhanced GAN for signal denoising with a complex-valued CNN for accurate spatial feature extraction, addressing key challenges in conventional and deep learning-based DOA estimation methods. The GAN component, equipped with a phase-consistent loss function, effectively suppresses noise while preserving spatial phase information essential for accurate direction finding. The subsequent complex-valued CNN processes enhanced covariance matrices to extract discriminative spatial features for precise DOA classification.

Extensive experimental evaluations demonstrate the superior performance of the proposed framework across a wide range of SNR conditions, snapshot numbers, and non-Gaussian noise environments. Specifically, the method achieves a DOA accuracy of 72.2% and an RMSE of 3.9° at —10 dB SNR with 500 snapshots, significantly outperforming traditional algorithms such as MUSIC and ESPRIT, as well as state-of-the-art deep learning baselines. The framework also exhibits strong robustness to limited data, maintaining 93.8% accuracy with only 50 snapshots at 20 dB SNR. Furthermore, the proposed approach demonstrates consistent performance under various non-Gaussian noise conditions, highlighting its practical applicability in real-world scenarios.

The results validate the effectiveness of the integrated enhancement-estimation paradigm and underscore the importance of phase-preserving denoising in DOA estimation. Future work will focus on extending the framework to more complex array geometries, exploring online adaptation mechanisms for dynamic environments, and investigating lightweight architectures for real-time deployment on embedded systems.

## Figures and Tables

**Table 1:** SNR improvement comparison with 500 snapshots (dB).

Method	—10 dB	—5 dB	0 dB	10 dB	20 dB
Wavelet Denoising	1.8	2.1	2.8	3.5	3.8
Spectral Subtraction	2.9	3.2	3.9	4.6	4.9
Kalman Filter	3.7	4.1	4.8	5.4	5.7
Proposed GAN	6.2	6.8	7.2	7.8	8.1

**Table 2:** DOA estimation accuracy comparison under different SNR conditions with 500 snapshots (%).

Method	—10 dB	—5 dB	0 dB	10 dB	20 dB
MUSIC	15.2	24.5	48.3	82.8	95.5
ESPRIT	13.8	22.1	50.7	85.6	96.8
CNN-Based	62.4	72.6	85.8	92.7	97.2
ResNet-Based	65.7	76.3	86.9	93.5	97.8
GAN-CNN	72.2	87.4	92.7	95.9	98.9

**Table 3:** DOA estimation accuracy under different snapshot numbers at 20 dB SNR (%).

Method	50	80	100	200	500
MUSIC	71.3	76.2	81.4	89.7	95.5
ESPRIT	68.5	78.9	83.6	91.2	96.8
CNN-Based	89.2	91.8	93.5	95.8	97.2
ResNet-Based	90.6	92.7	94.1	96.3	97.8
GAN-CNN	93.8	95.2	96.1	97.6	98.9

**Table 4:** RMSE comparison under different SNR conditions with 500 snapshots (degrees).

Method	—10 dB	—5 dB	0 dB	10 dB	20 dB
MUSIC	12.8	9.6	5.8	2.3	1.1
ESPRIT	13.5	10.2	6.1	2.1	0.9
CNN-Based	5.2	3.8	2.4	1.3	0.7
ResNet-Based	4.7	3.4	2.1	1.1	0.6
GAN-CNN	3.9	2.6	1.5	0.8	0.4

**Table 5:** RMSE under different snapshot numbers at 20 dB SNR (degrees).

Method	50	80	100	200	500
MUSIC	4.2	3.1	2.6	1.8	1.1
ESPRIT	3.8	2.8	2.3	1.5	0.9
CNN-Based	1.8	1.4	1.2	0.9	0.7
ResNet-Based	1.5	1.2	1.0	0.8	0.6
GAN-CNN	1.1	0.9	0.8	0.6	0.4

**Table 6:** DOA estimation accuracy under different non-Gaussian noise conditions at 10 dB SNR with 500 snapshots (%).

Method	Gaussian Noise	Laplacian Noise	Uniform Noise	Mixture Gaussian
MUSIC	82.8	65.2	70.8	75.4
ESPRIT	85.6	68.4	73.1	78.2
CNN-Based	92.7	81.3	85.9	88.6
ResNet-Based	93.5	83.7	87.4	90.1
GAN-CNN	95.9	89.2	91.8	93.7

**Table 7:** Training time comparison across different methods.

Method	Training Time (s)
MUSIC	N/A (no training)
ESPRIT	N/A (no training)
CNN-Based	312.6
ResNet-Based	498.3
Proposed GAN–CNN	673.9

## Data Availability

The data will be made available by the authors upon request.

## Abbreviations

The following abbreviations are used in this manuscript:

DOA	Direction of Arrival
SNR	Signal-to-Noise Ratio
GAN	Generative Adversarial Network
CNN	Convolutional Neural Network
MUSIC	Multiple Signal Classification
ESPRIT	Estimation of Signal Parameters via Rotational Invariance Techniques
RMSE	Root Mean Square Error
ULA	Uniform Linear Array
STFT	Short-Time Fourier Transform
